# Captopril Pretreatment Produces an Additive Cardioprotection to Isoflurane Preconditioning in Attenuating Myocardial Ischemia Reperfusion Injury in Rabbits and in Humans

**DOI:** 10.1155/2015/819232

**Published:** 2015-07-27

**Authors:** Yi Tian, Haobo Li, Peiyu Liu, Jun-mei Xu, Michael G. Irwin, Zhengyuan Xia, Guogang Tian

**Affiliations:** ^1^Department of Anesthesiology, Haikou Municipal Hospital, Affiliated Haikou Hospital Xiangya School of Medicine, Central South University, Haikou 570208, China; ^2^Department of Anesthesiology, The University of Hong Kong, Hong Kong; ^3^Department of Anesthesiology, Hainan Municipal Corps Hospital, Chinese People's Armed Police Force, Haikou 570203, China; ^4^Department of Anesthesiology, The Second Xiangya Hospital, Central South University, Changsha, Hunan 410011, China; ^5^Department of Anesthesiology, Affiliated Hospital of Guangdong Medical College, Zhanjiang 524023, China

## Abstract

*Background.* Pretreatment with the angiotensin-converting inhibitor captopril or volatile anesthetic isoflurane has, respectively, been shown to attenuate myocardial ischemia reperfusion (MI/R) injury in rodents and in patients. It is unknown whether or not captopril pretreatment and isoflurane preconditioning (Iso) may additively or synergistically attenuate MI/R injury.* Methods and Results.* Patients selected for heart valve replacement surgery were randomly assigned to five groups: untreated control (Control), captopril pretreatment for 3 days (Cap3d), or single dose captopril (Cap1hr, 1 hour) before surgery with or without Iso (Cap3d+Iso and Cap1hr+Iso). Rabbit MI/R model was induced by occluding coronary artery for 30 min followed by 2-hour reperfusion. Rabbits were randomized to receive sham operation (Sham), MI/R (I/R), captopril (Cap, 24 hours before MI/R), Iso, or the combination of captopril and Iso (Iso+Cap). In patients, Cap3d+Iso but not Cap1hr+Iso additively reduced postischemic myocardial injury and attenuated postischemic myocardial inflammation. In rabbits, Cap or Iso significantly reduced postischemic myocardial infarction. Iso+Cap additively reduced cellular injury that was associated with improved postischemic myocardial functional recovery and reduced myocardial apoptosis and attenuated oxidative stress.* Conclusion.* A joint use of 3-day captopril treatment and isoflurane preconditioning additively attenuated MI/R by reducing oxidative stress and inflammation.

## 1. Introduction

Myocardial ischemia reperfusion (MI/R) injury is a major perioperative complication that is associated with significant morbidity and mortality in coronary artery bypass graft (CABG) surgery [1, 2] and in patients undergoing heart valve replacement surgery [3] using cardiopulmonary bypass (CPB), especially in patients with comorbidities (e.g., age [4] and diabetes [5]). Volatile anesthetic preconditioning (e.g., isoflurane preconditioning, Iso) provides cardioprotective effects during CABG [6, 7]. Given that the isoflurane preconditioning cardioprotection is optimal or effective only when duration of the index ischemia is limited within 25–40 minutes [8] while the typical period of cardiac ischemia during CABG surgery is usually longer than 60 minutes, the cardioprotective potential of Iso in the clinical settings is limited. Moreover, in aging [9] and diabetes [10], in which myocardial oxidative stress and inflammation are increased concomitant with reduced endogenous antioxidant capacity, cardioprotection of Iso is diminished or abolished.

Increased systemic oxidative stress induced by robust production of reactive oxygen species (ROS) has been suggested as the main cause which adversely affects postoperative cardiac functional recovery in patients undergoing CABG surgery using CPB [11]. ROS not only increases oxidative stress by increasing lipid peroxidation but also reduces myocardial antioxidant capacity by diminishing endogenous antioxidant enzymes activities (e.g., superoxide dismutase) [12, 13]. In addition, proinflammatory cytokines release (e.g., tumor necrosis factor- (TNF-) *α*) is also enhanced after CPB [14], which may further increase ROS production and exacerbate myocardial function [15]. Experimental studies show that Iso confers cardioprotection against myocardial IRI by stimulating the generation of small amount of ROS which triggers cardiac protective signaling pathways [16, 17]. On the other hand, experimental studies showed that volatile anesthetic preconditioning exert cardioprotective effects against myocardial ischemic reperfusion injury through reducing nuclear factor- (NF-) kB-dependent inflammatory gene expression and decreasing TNF-*α* production [18, 19].

Captopril, an angiotensin-converting enzyme (ACE) inhibitor, has been shown to be cardioprotective in the prevention and regression of left ventricular hypertrophy or attenuation of MI/R injury in both clinical [20] and experimental settings [21]. Captopril pretreatment exerts cardioprotective effects by increasing tissue antioxidant activity, scavenging different types of ROS, and thus prevents lipid peroxidation [22, 23]. Captopril by inhibiting angiotensin-converting enzyme activity reduced the degradation of bradykinin, resulting in enhanced formation of prostacyclin and nitric oxide, and in turn increases myocardial antioxidant and anti-inflammatory properties [20, 24] and confers cardioprotective effects [25]. Therefore, the mechanism of captopril cardioprotection is totally different from that of isoflurane preconditioning. Thus, it is plausible that captopril in combination with isoflurane preconditioning may synergistically or additively attenuate MI/R in clinical settings. Therefore, we hypothesized that alternative use of captopril pretreatment and isoflurane preconditioning may confer superior protection against MI/R injury to either isoflurane or captopril regimen alone and that the mechanism of the additive/synergistic effect is related to reducing cardiomyocytes apoptosis and myocardial oxidative stress.

## 2. Methods

### 2.1. Patient Population and Study Design

The clinical trial was carried out in accordance with the Declaration of Helsinki (2000) of the World Medical Association. The study protocol was approved by the institutional ethics committee. All subjects gave written informed consent after having been given full explanation of the purpose, nature, and risk of all procedures used.

After obtaining written informed consent, 100 ASA (American Association of Anesthesiologists) class II to III patients, aged 38–55 years, presenting for scheduling for heart mitral valve replacement surgery were assigned according to a computer-generated random code to one of the five groups: a control group receiving midazolam and fentanyl (group control; *n* = 20); captopril pretreatment for 1 hour (Cap1hr, 12.5 mg, oral administration) or 3 days (Cap3d, 12.5 mg, 3 times per day, oral administration) before surgery groups (*n* = 20 per group); and a combination of isoflurane preconditioning (Iso) and captopril pretreatment (1 hour or 3 days before surgery) (group Cap1hr+Iso or Cap3d+Iso; *n* = 20 per group). Subjects were assigned treatment numbers in ascending chronological order of admission in the study. The surgeons, research assistants, and medical and nursing staff in the operation room were blinded to the group assignments, facilitated by covering the drug infusion pump and lines and shielding the isoflurane vaporizer from view.

The exclusion criteria were (a) cardiogenic shock, (b) left main coronary artery occlusion or severe stenosis, (c) blood flow in the infarct-related artery > thrombolysis in myocardial infarction grade 1, (d) treatment with glycoprotein IIb/IIIa receptor antagonists before the procedure, and (e) infection or surgery within 2 weeks [6, 26].

### 2.2. Anesthetic Protocols and Surgery

**Figure 1:**
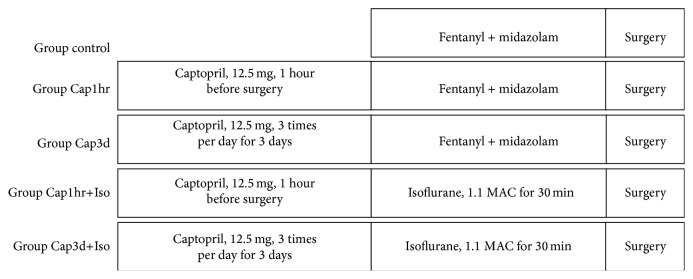
Protocol used in experimental groups. Patients were pretreated without or with captopril for 1 hour (Cap1hr, 12.5 mg, oral administration) or 3 days (Cap3d, 12.5 mg, 3 times per day, oral administration) before surgery. After induction, all patients received continuous infusions of fentanyl at 0.6 *μ*g·kg^−1^ of body weight·min^−1^ and pancuronium bromide at 15 *μ*g·kg^−1^ of body weight·min^−1^ during surgery. The anaesthesia protocol in various groups is as follows: anaesthesia was maintained either with fentanyl and midazolam (group control, Cap1hr, and Cap3d) or with isoflurane 1.1 MAC (minimum alveolar anesthetic concentration) end tidal before surgery (group Cap1hr+Iso or Cap3d+Iso).

All patients received standard premedication of scopolamine at 0.006 mg/kg of body weight and morphine at 0.1 mg/kg of body weight intramuscularly 60 min before surgery. In all groups, anaesthesia was induced with etomidate at 0.3 mg/kg of body weight, fentanyl at 8 *μ*g/kg of body weight, and pancuronium bromide at 0.1 mg/kg of body weight given intravenously. After induction, all patients received continuous infusions of fentanyl at 0.6 *μ*g·kg^−1^ of body weight·min^−1^ and pancuronium bromide at 15 *μ*g·kg^−1^ of body weight·min^−1^ during surgery. The anaesthesia protocol in various groups is as follows (see Figure 1): anaesthesia was maintained either with fentanyl and midazolam (group control, Cap1hr, and Cap3d) or with isoflurane 1.1 MAC (minimum alveolar anesthetic concentration) end tidal before surgery (group Cap1hr+Iso or Cap3d+Iso). During anaesthesia, patients were monitored with five-lead ECG, pulse oximetry, capnography, invasive arterial pressure, and pulmonary artery pressure during the operation.

Central venous blood samples were obtained prior to CPB (Baseline), 20 min after CPB induction, 30 min, 4 h, and 24 h after aortic declamping for the measurements of plasma levels of cTnI (cardiac troponin I) and CK-MB (creatine kinase MB), TNF- (tumor necrosis factor-) *α*, IL- (interleukin-) 6, and ICAM (intercellular adhesion molecule). Samples were immediately cooled to 4°C and centrifuged at 1000 g for 10 min at 4°C. Plasma was collected and stored at −70°C until analyzed.

### 2.3. Animal Study Experimental Protocol

The study was approved by the institutional ethic committee and conforms with U.S. National Institutes of Health guidelines. Adult New Zealand white rabbits (1.8 kg) were anaesthetized with sodium pentobarbital (30 mg/kg, Alfasan, Holland). The rabbits were ventilated with 100% oxygen and end tidal PCO_2_ were maintained between 35 and 45 mmHg. Myocardial ischemia reperfusion (I/R) was produced by exteriorizing the heart through a left thoracic incision and occluding the LAD (left anterior descending artery) with a silk slipknot. After 40 min of ischemia, the slipknot was released and the myocardium was reperfused for 4 hrs. Sham (sham operated control rabbits) underwent the same surgical procedures except that the LAD was not occluded.

Rabbits were randomized to receive one of the following treatments (*n* = 8 each): Sham, rabbits receiving vehicle (0.9% NaCl) without being subjected to I/R; I/R, rabbits receiving vehicle during reperfusion; IPC, rabbits receiving IPC (3 cycles of 10 s of coronary artery reocclusion and reperfusion) before ischemia; Iso, rabbits receiving isoflurane (15 min 1.1% end tidal isoflurane followed by a 15 min washout period) before inducing ischemia; Cap, rabbits receiving captopril (25 mg/kg, oral administration) 24 hours before inducing ischemia; Iso+Cap, rabbits receiving isoflurane in combination with captopril before inducing ischemia.

### 2.4. Determination of Myocardial Functional Recovery in Rabbits

In the rabbits, LV function was continuously monitored during the entire MI/R period via a Millar Mikro-Tip catheter pressure transducer inserted into the LV via left carotid artery as we have described previously. HR (heart rate), MAP, and RPP were derived by computer algorithms (Chengdu Instrument).

### 2.5. Determination of Postischemic Myocardial Infarct Size in Rabbits and Cellular Injury

The infarct size was measured with a double-staining technique using Evans Blue-TTC (Triphenyltetrazolium Chloride) staining and a digital imaging system, as described previously (additional *n* = 8 rabbits per group were used for infarct size determination). Blood samples were drawn before ligation and at the end of reperfusion. Plasma cTnI (cardiac-specific troponin I) from rabbits and patients were measured spectrophotometrically (Backman DU 640 instrument) with commercially available assay kits (Nanjing Jiancheng). The plasma samples were coded, and the levels of cTnI (cardiac specific troponin I) were assayed in duplicate by an investigator initially blinded to the research groups. Plasma IL-6, ICAM, TNF-*α*, and CK-MB were measured using ELISA kits (R&D Systems). Plasma samples used for biochemical assays were coded, and the laboratory investigator was blinded in regard to treatment regimen. All haemodynamic data were collected by trained observers who did not take an authorship in this study and who were blinded to the methods regarding anaesthetic and captopril usage.

### 2.6. Assessment of Rabbit Myocardial Apoptosis by Flow Cytometry Analysis

At the end of reperfusion, hearts were removed and washed twice with ice-cold phosphate-buffered saline; cell suspensions were prepared using the Medimachine System (Becton-Dickinson). Cardiomyocyte cells were collected by flow cytometry with the use of an antibody to a-sarcomeric actin. The cells were resuspended in binding buffer, after adding FITC-Annexin V and propidium iodide, the mixture was incubated for 10 minutes in the dark at 4°C, and then cellular fluorescence was measured with a fluorescence activated cell sorter scan flow cytometer (Becton, Dickinson and Company, Franklin Lakes, NJ) as described [27].

### 2.7. Determination of MDA (Malondialdehyde) and SOD (Superoxide Dismutase) in Rabbit

MDA is end product of the ROS- (reactive oxygen species-) mediated lipid peroxidation cascade. Rabbit blood samples were collected, and the MDA levels were measured with a commercial kit (Baster Biological Tech), expressed as nmol/mL. SOD activity was detected in cardiac tissue homogenates using commercially available kit (Cayman Chemical) as described previously, expressed as U/mL.

### 2.8. Western Blot Analysis

Equal amount of proteins from rabbit heart homogenate was resolved by 7.5–15% sodium dodecyl sulfate polyacrylamide gel electrophoresis and transferred to nitrocellulose membranes and processed as described. The primary antibodies against BCL-2, Bax, and GAPDH were purchased from Cell Signaling Technology (Beverly, MA). Immunoreactive bands were visualized by enzymatic chemiluminescence method and quantified with Quantity One image software.

### 2.9. Sample Size Estimation

Group sample size was estimated based on differences in cTnI concentration measured at 4 h after CPB in a pilot study of patients who received isoflurane (1–1.5 minimum alveolar concentration throughout the surgery) anaesthesia. The formula *n* = 15.7/ES^2^ + 1, where ES = effect size = (difference between groups)/(mean of the S.D. between groups), with *α* = 0.05 and power = 0.8, was used to determine that the study would be adequately powered with *n* = 20 per group.

### 2.10. Statistical Analysis

All continuous data are expressed as means ± SD. Statistical evaluation of patients' file and perioperative data was performed by unpaired Student's *t*-test or *χ* ^2^ test when appropriate. Between-groups and within-group differences of bioassay data were analyzed using two-way ANOVA with repeated measures and Bonferroni's corrections (Graphpad Prism). Values were considered statistically significant when *P* < 0.05.

## 3. Results

### 3.1. Human Study Data

#### 3.1.1. Preoperative and Intraoperative Data in Patients

As shown in Table 1. The patients' baseline demographics, clinical measures of cardiac function, and medications (digoxin and diuretic) did not differ among groups. As shown in Table 2, the operation time, aortic clamping time, and bypass time did not differ among groups. Automatic rebeating rate in groups Cap3d+Iso and Cap1hr+Iso was significantly higher than that in control group, while no significant difference was observed in groups Cap3d and Cap1hr as compared to control group. Arrhythmia incidence was significantly higher in control group than that in all treatment groups (Cap3d+Iso, Cap1hr+Iso, Cap3d, and Cap1hr) and there is no difference among all treatment groups. Similarly, dopamine usage was significantly reduced in all treatment groups compared with the control groups and there was no difference among all treatment groups. Sodium nitroprusside usage was significantly reduced in all treatment groups compared with the control groups and sodium nitroprusside usage was significantly reduced in group Cap3d+Iso relative to other groups.

#### 3.1.2. Postoperative Outcome Data in Patients

As shown in Table 2, durations of postoperative ICU stay in all treatment groups were shorter than that in the control group, and durations of postoperative ICU stay in groups Cap3d+Iso and Cap1hr+Iso were significantly shorter than that in groups Cap3d and Cap1hr. The duration of hospital stay in group Cap3d+Iso, but not groups Cap3d or Cap1hr, was shorter than that in Cap1hr+Iso and the duration of hospital stay in all treatment was significantly reduced compared with control group. All patients were discharged from hospital uneventfully.

#### 3.1.3. Isoflurane and Captopril Attenuated Postischemic Myocardial Cellular Injury and Reduced Proinflammatory Cytokines TNF-*α*, IL-6, and ICAM in Patients

**Figure 2:**
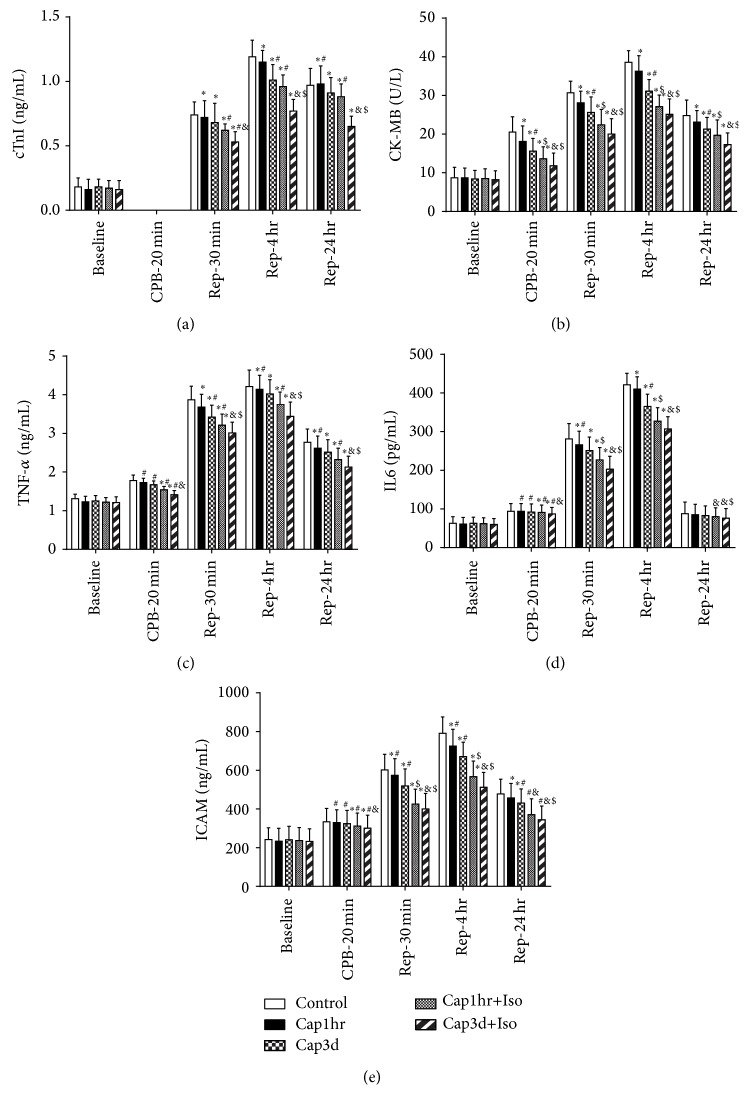
Isoflurane (Iso) and captopril (Cap) attenuated postischemic myocardial cellular injury and reduced proinflammatory cytokines release in patients. (a) Plasma cardiac troponin I (cTnI), (b) plasma creatine kinase- (CK-) MB, (c) plasma tumor necrosis factor- (TNF-) *α*, (d) plasma interleukin- (IL-) 6, and (e) plasma intercellular adhesion molecule (ICAM). Control: untreated control; Cap1hr: captopril treatment for 1 h before surgery (12.5 mg, p.o., 1 h before surgery); Cap3d: captopril treatment for 3 days (12.5 mg, p.o., 3 times a day) before surgery; Cap1hr+Iso: captopril treatment for 1 h in combination with inhalational isoflurane (Iso) at 1.1% end tidal concentration administrated for 30 min before CPB; Cap3d+Iso: captopril treatment for 3 days in combination with Iso before CPB. Data are shown as means ± SD, with *n* = 20 per group. ^#^ *P* < 0.05 versus Baseline, ^*^ *P* < 0.05 and ^$^ *P* < 0.01 versus Control, and ^&^ *P* < 0.05 versus Cap1hr+Iso.

Baseline plasma levels of cTnI, CK-MB, TNF-*α*, IL-6, and ICAM did not differ among groups, but all significantly increased in control group both during CPB and during reperfusion at 30 minutes and 4 hours after CPB (Figures 2(a)–2(e)), while post-CPB plasma levels of CK-MB, TNF-*α*, IL-6, and ICAM did not differ from that in baseline (Figures 2(b)–2(e)). During CPB and during reperfusion at 30 minutes, 4 hours, and 24 hours after CPB, plasma levels of cTnI, CK-MB, TNF-*α*, IL-6, and ICAM in Cap3d+Iso, Cap1hr+Iso, and Cap3d, but not in Cap1hr, were significantly lower than that in the control groups, and those in group Cap3d+Iso and Cap1hr+Iso were significantly lower than that in other groups (Figures 2(a)–2(e)). Plasma levels of cTnI during CPB and during reperfusion at 30 minutes, 4 hours, and 24 hours after CPB in group Cap3d+Iso were significantly lower than that in Cap1hr+Iso (Figure 2(a)).

### 3.2. Animal Study Data

#### 3.2.1. Isoflurane and Captopril Reduced Postischemic Myocardial Injury, Ameliorated Myocardial Inflammation, and Attenuated Cardiac Ultrastructural Alterations in Rabbits

**Figure 3:**
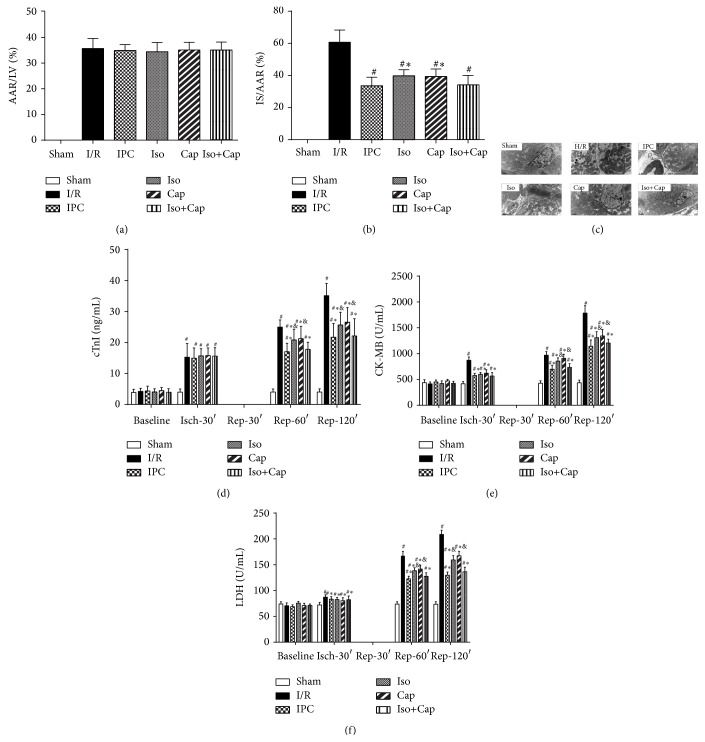
Isoflurane (Iso) and captopril (Cap) reduced postischemic myocardial injury, ameliorated myocardial inflammation, and attenuated cardiac ultrastructural alterations in rabbits. (a) Ratio of area at risk (AAR) to left ventricular (LV), (b) percentage of infarct size (IS) expressed as a percentage of AAR, (c) representative images of electron micrograph of rabbit hearts, (d) plasma cTnI, (e) plasma CK-MB, and (f) lactate dehydrogenase (LDH). Sham: sham operated control; I/R: rabbits subjected to myocardial ischemia reperfusion (MI/R); Cap: rabbits treated with captopril (25 mg/kg given intravenously 24 h) prior to inducing MI/R; Iso: rabbits received isoflurane preconditioning (15 min 1.1% end tidal isoflurane followed by a 15 min washout period before inducing MI/R); Iso+Cap: rabbits received captopril in combination with Iso. Data are shown as means ± SD, with *n* = 8 animals per group. ^#^ *P* < 0.05 or *P* < 0.01 versus I/R, ^*^ *P* < 0.05 versus IPC, and ^&^ *P* < 0.05 versus Baseline.

As shown in Figure 2, the myocardial area at risk did not differ among groups (Figure 3(a)). I/R resulted in significantly increased myocardial IS in I/R group. IPC, Iso, or Cap alone significantly reduced IS, while Cap in combination with Iso (Cap+Iso group) yielded an additive effect in that they further decreased IS as compared with either Iso or Cap alone (Figure 3(b)). Biopsies taken from sham operated control rabbit heart showed basically normal ultrastructure, with regular intercalated disks, sarcomere preservation, and normal mitochondrial morphology, although mild cytosolic, intermyofibrillar edema, and nuclear chromatin margination could occasionally be seen. In contrast, biopsies taken after postischemic reperfusion from rabbit heart showed signs of injury; in particular, cytosolic and intermyofibrillar edema was moderate to marked, and mitochondria showed more extensive damage compared with that in sham operated group. Biopsies taken from IPC, Cap, Iso, and Cap+Iso groups showed normal myofibrillar ultrastructure and mild separation of the mitochondrial cristae without swelling and vacuolation (Figure 3(c)).

#### 3.2.2. Isoflurane and Captopril Improved Postischemic Myocardial Function Recovery in Rabbits

**Figure 4:**
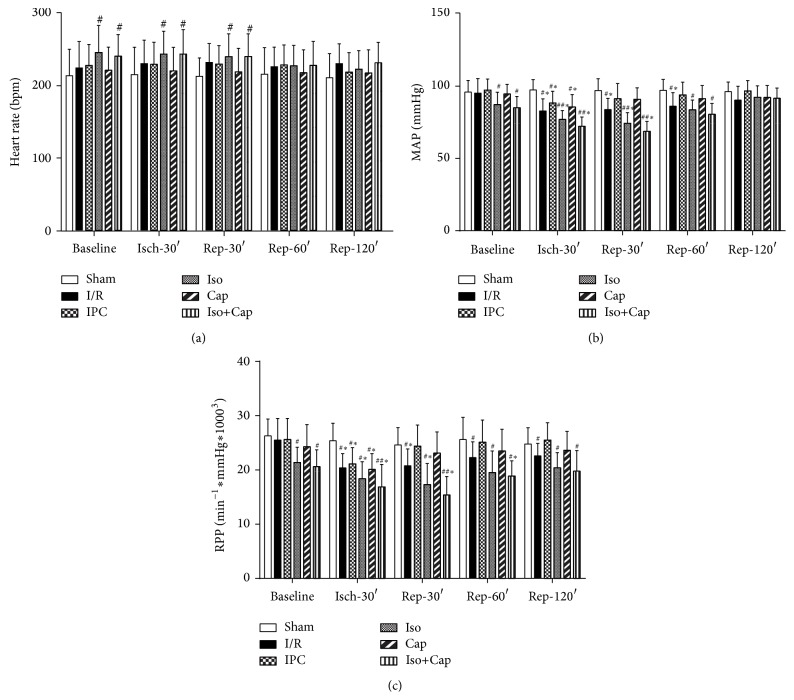
Isoflurane (Iso) and captopril (Cap) improved postischemic myocardial function recovery in rabbits. (a) Heart rate, (b) mean arterial pressure (MAP), and (c) rate pressure product (RPP) at baseline and during ischemia and reperfusion. Sham: sham operated control; I/R: rabbits subjected to myocardial ischemia reperfusion (MI/R); Cap: rabbits treated with captopril (25 mg/kg given intravenously 24 h) prior to inducing MI/R; Iso: rabbits received isoflurane preconditioning (15 min 1.1% end tidal isoflurane followed by a 15 min washout period before inducing MI/R); Iso+Cap: rabbits received captopril in combination with Iso. Data are shown as means ± SD, with *n* = 8 animals per group. ^#^ *P* < 0.05 or *P* < 0.01 versus Sham, ^*^ *P* < 0.05 versus Baseline.

As shown in Figure 3, heart rate in groups Iso and Cap+Iso was significantly higher than that in other groups at baseline, and during ischemia, and during reperfusion at reperfusion 30 minutes, respectively, while no significant difference was observed among groups during reperfusion at reperfusion 60 minutes and 120 minutes (Figure 4(a)). Mean arterial pressure (MAP) in groups Iso and Cap+Iso was significantly lower than that in other groups and did not differ between groups in baseline, ischemia for 30 minutes, reperfusion for 30 minutes, and reperfusion for 60 minutes, and no significant differences of MAP were observed among groups (Figure 4(b)). Rate pressure product (RPP) in groups Iso and Iso+Cap was significantly lower than that in other groups at baseline, during I/R, and reperfusion, while there was no difference between Iso and Iso+Cap groups (Figure 4(c)).

#### 3.2.3. Isoflurane and Captopril Attenuated Postischemic Myocardial Apoptosis in Rabbits

**Figure 5:**
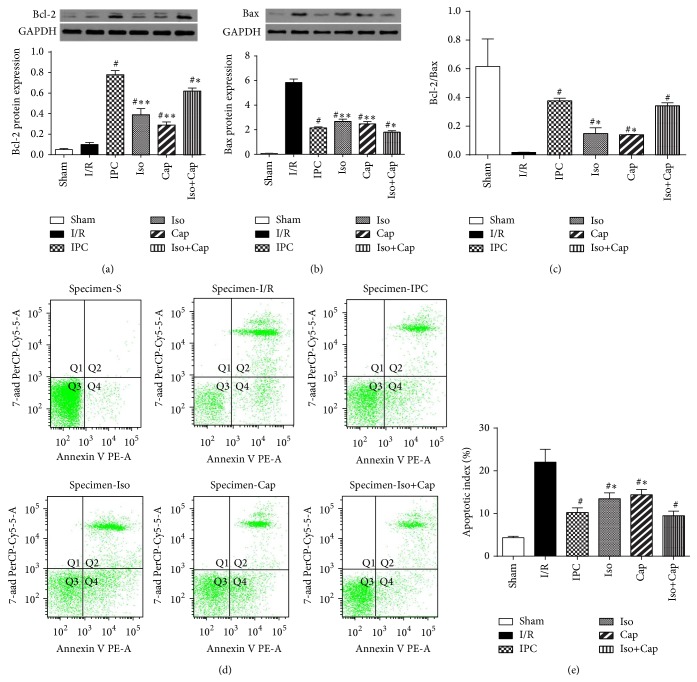
Isoflurane (Iso) and captopril (Cap) attenuated postischemic myocardial apoptosis in rabbits. (a) Protein expression of cardiac Bcl-2, (b) protein expression of cardiac Bax, (c) ratio of Bcl-2 to Bax, (d) representative graphs showing the proportion of apoptotic myocytes, and (e) quantitation of apoptotic myocytes. Sham: sham operated control; I/R: rabbits subjected to myocardial ischemia reperfusion (MI/R); Cap: rabbits treated with captopril (25 mg/kg given intravenously 24 h) prior to inducing MI/R; Iso: rabbits received isoflurane preconditioning (15 min 1.1% end tidal isoflurane followed by a 15 min washout period before inducing MI/R); Iso+Cap: rabbits received captopril in combination with Iso. Data are shown as means ± SD, with *n* = 8 animals per group. ^#^ *P* < 0.05 versus I/R, ^*^ *P* < 0.05 versus IPC.

Bcl-2, an antiapoptotic protein, was moderately increased in I/R groups but was significantly increased in IPC, Iso, Cap, and Iso+Cap groups, and Bcl-2 in IPC and Iso+Cap groups were significantly higher than that in Iso or Cap groups (Figure 5(a)). Proapoptotic protein Bax was significantly increased in I/R and significantly reduced in IPC, Iso, Cap, and Iso+Cap groups, and there was no significant difference among IPC, Iso, Cap, and Iso+Cap groups (Figure 5(b)). Bcl-2/Bax ratio was significantly reduced in I/R group compared with Sham group. Bcl-2/Bax ratio was enhanced by IPC, Iso, Cap, and Iso+Cap as compared to control, and IPC and Iso+Cap significantly further increased Bcl-2/Bax ratio compared with Iso or Cap alone (Figure 5(c)); the respective protein expressions were further confirmed by flow cytometry (Figures 5(d) and 5(e)).

#### 3.2.4. Isoflurane and Captopril Ameliorated Postischemic Myocardial Oxidative Stress in Rabbits

**Figure 6:**
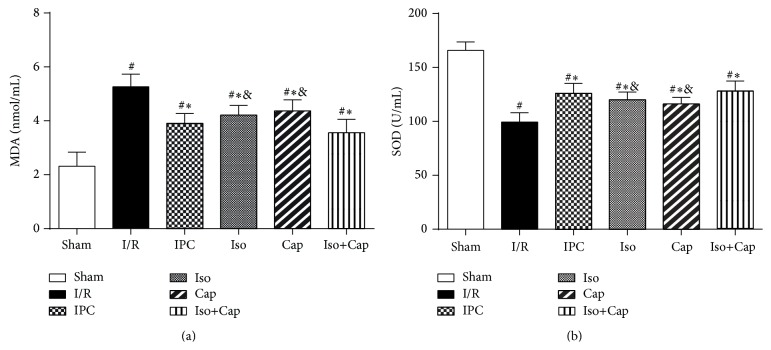
Isoflurane (Iso) and captopril (Cap) ameliorated postischemic myocardial oxidative stress in rabbits. (a) Cardiac malondialdehyde (MDA) content, (b) plasma superoxide dismutases (SOD) levels. Sham: sham operated control; I/R: rabbits subjected to myocardial ischemia reperfusion (MI/R); Cap: rabbits treated with captopril (25 mg/kg given intravenously 24 h) prior to inducing MI/R; Iso: rabbits received isoflurane preconditioning (15 min 1.1% end tidal isoflurane followed by a 15 min washout period before inducing MI/R); Iso+Cap: rabbits received captopril in combination with Iso. Data are shown as means ± SD, with *n* = 8 animals per group. ^#^ *P* < 0.05 versus Sham, ^*^ *P* < 0.05 versus I/R, and ^&^ *P* < 0.05 versus IPC.

MDA, an index of lipid peroxidation, was significantly increased in I/R and was moderately reduced in IPC, Iso, Cap, and Iso+Cap groups relative to I/R groups (Figure 6(a)). Plasma SOD in I/R group was significantly lower as compared to sham operative group, and IPC, Iso, Cap, and Iso+Cap slightly increased SOD as compared to I/R but the difference did not reach statistical difference (Figure 6(b)).

## 4. Discussion

The novel finding of the present clinical and animal studies is that the application of 3-day captopril treatment, but not 1-hour captopril treatment, in combination with isoflurane preconditioning before prolonged index ischemia was superior to either captopril treatment (3 days or 1 hour) or isoflurane alone in reducing postischemic myocardial oxidative stress, proinflammatory cytokines release, and myocardial injury. Combinational use of captopril as well as isoflurane preconditioning confers antioxidative stress and anti-inflammatory effects not only during ischemia but also during reperfusion stage in patients undergoing CPB. To our knowledge, this is the first study to demonstrate that captopril and isoflurane preconditioning can additively attenuate myocardial ischemia reperfusion (MI/R) injury.

When ischemia occurs, the oxygen and nutrient supply to the myocardium is reduced due to the decrease of blood flow. This deprivation of oxygen as well as nutrient supply results in the accumulation of sodium, hydrogen, and calcium ions, culminating in tissue acidosis, which leads to mitochondrial membrane depolarization, ATP depletion, and inhibition of myocardial contractile function [28]. Reperfusion, in turn, elicits rapid alterations in ion flux, stimulates a robust formation of ROS [29], and promotes the proinflammatory cytokines release [30], which eventually results in or exacerbates MI/R injury [31, 32]. Modulation of the early events induced by ischemia is of particular importance in combating MI/R. In the present study, in patients with CPB, a joint use of captopril and isoflurane preconditioning significantly reduced MI/R-induced proinflammatory cytokines (TNF-*α*, IL-6, and ICAM-1) release during CPB, which was associated with reduced myocardial injury during CPB and after CPB (reperfusion), and this protective effect of captopril in combination of isoflurane preconditioning is superior to captopril treatment for 3 days or 1 hour alone. Solenkova et al. reported that, in isolated rabbit heart model, cardioprotection of ischemic preconditioning is dependent on activation of adenosine receptors during the first minutes of reperfusion following termination of the index ischemia [33]. Similarly, Kin et al. showed that the first minute of the onset of reperfusion is critical to the cardioprotective effects of ischemic postconditioning, which protects the heart against MI/R injury by inhibiting oxidant generation and oxidant mediated injury in rats [34]. These together with the results from our present study jointly support the notion that inhibition of myocardial oxidative stress and inflammation occurred during ischemia or in the early minutes at the onset of reperfusion is important for developing cardioprotective strategies against MI/R. Results from our present study provide a clue that inhibition of myocardial oxidative stress and inflammation as early as during ischemia plays a pivotal role in ameliorating myocardial injury in MI/R. Of note, in animal study, captopril, isoflurane, and their combination all mimic the protective effects of IPC in reducing myocardial injury during ischemia, while there is no significant difference observed among all treatment groups (IPC, Cap, Iso, and Iso+Cap). This is not consistent with the results seen in our human study which showed that additive effects of captopril and isoflurane preconditioning exist as early as ischemia induction (CPB). One possible explanation is that the severity of MI/R injury is different between patients with CPB and animals received MI/R in the current study and that captopril treatment for 24 hours in animals may not precisely mimic that in our human study (3 days or 1 hour before CPB).

Increased oxidative stress and evaluated inflammation have been suggested as the main cause of MI/R injury. We previously showed that antioxidant N-acetylcysteine and allopurinol confer cardioprotective effects in rat subjected to MI/R [29, 35] and isoflurane preconditioning and propofol postconditioning synergistically reduced MI/R injury in patients undergoing CPB by upregulating endothelial nitric oxide synthase protein expression and reducing oxidative stress and proinflammatory cytokines release [6]. Similarly, in the present study, in animal model of MI/R, captopril, isoflurane preconditioning, and their combination all reduced MDA (marker of lipid peroxidation) and increased SOD activity, which were accompanied with decreased myocyte apoptosis and reduced myocardial injury and inflammation. Captopril in combination with isoflurane preconditioning confers superior protection to either captopril or isoflurane alone in rabbits subjected to MI/R. However, in patients with CPB, captopril (3 days or 1 hour) in combination with isoflurane preconditioning and captopril treatment alone, for 3 days but not 1 hour, reduced MI/R injury and mycocardial inflammation. This inconsistence suggests that captopril cardioprotective effects maybe depend on the duration of the treatment. van den Heuvel et al. demonstrated that captopril treatment for 3 months reduces the incidence of ischemia-related events after myocardial infarction, but a high incidence of clinical events occurred with withdrawal from captopril after 3-month treatment relative to 1-year captopril treatment [36]. This is consistent with our present finding that no cardioprotective effect was observed in patients treated with captopril 1 hour before CPB, while 3-day captopril treatment exerted profound protection. It is of notice that, although captopril treatment for 1 hour alone did not show a protective effect in patients underwent CPB in the current study, captopril treatment for 1 hour in combination of isoflurane preconditioning conferred cardioprotective effects, indicating that captopril treatment may facilitate isoflurane preconditioning to confer protection. Captopril by inhibiting angiotensin-converting enzyme activity reduces the degradation of bradykinin, resulting in enhanced formation of prostacyclin and nitric oxide (NO), consequently exerting its antioxidant and anti-inflammatory properties [20, 24] and conferring cardioprotective effects [25]. NO has been proven as a trigger and mediator of isoflurane preconditioning cardioprotection. Isoflurane preconditioning by increasing endothelial nitric oxide synthase (NOS), but not inducible or neuronal NOS, enhances NO production and confers cardioprotection in a rabbit MI/R model [37]. These together suggest that captopril and isoflurane preconditioning can confer additive/synergistic effects in combating MI/R. This is confirmed in our present clinical study and animal study. However, further study is needed to elucidate the specific molecular mechanism governing the synergy of captopril and isoflurane preconditioning in the setting of MI/R.

## 5. Conclusions

In summary, we demonstrated that isoflurane preconditioning, combined with captopril treatment (3 days but not 1 hour), acts additively in attenuating postischemic myocardial reperfusion injury as determined by the surrogate markers of myocardial injury and function. Further large-scale and long-term studies are required to confirm the clinical benefit of this novel pharmacologic regimen, which may offer a promising therapeutic approach to cardioprotection patients undergoing cardiac surgery.

## Figures and Tables

**Table 1:** Baseline characteristics of the study population.

Item	Control	Cap1hr	Cap3d	Cap1hr+Iso	Cap3d+Iso
Gender (male/female)	14/6	14/6	15/5	13/7	14/6
Age (years)	46.2 ± 5.9	46.8 ± 7.5	44.5 ± 6.9	43.2 ± 7.2	45.3 ± 6.1
Body weight (Kg)	58.2 ± 7.2	61.7 ± 8.1	62.1 ± 9.0	61.2 ± 7.9	59.7 ± 8.3
LVEF (%)	55.2 ± 6.9	55.3 ± 6.9	53.1 ± 7.8	54.2 ± 7.4	56.6 ± 6.3
Premedication					
Digoxin (*n*)	6	6	7	5	6
Diuretic (*n*)	8	7	6	7	8

Control: untreated control; Cap1hr: captopril treatment for 1 h before surgery (12.5 mg, p.o., 1 h before surgery); Cap3d: captopril treatment for 3 days (12.5 mg, p.o., 3 times a day) before surgery; Cap1hr+Iso: captopril treatment for 1 h in combination with inhalational isoflurane (Iso) at 1.1% end tidal concentration administrated for 30 min before CPB; Cap3d+Iso: captopril treatment for 3 days in combination with Iso before CPB. Values are means ± S.D. or the number of patients. No significant differences were observed among groups; LVEF: left ventricular ejection fraction.

**Table 2:** Intraoperative and postoperative procedure characteristics of the patients.

Item	Control	Cap1hr	Cap3d	Cap1hr+Iso	Cap3d+Iso
Operation time (min)	147 ± 43	147 ± 45	142 ± 41	144 ± 45	148 ± 42
Aortic clamping time (min)	46 ± 18	46 ± 17	45 ± 19	47 ± 16	43 ± 18
Bypass time (min)	65 ± 19	67 ± 19	68 ± 20	67 ± 22	68 ± 21
Automatic rebeating rate (%)	40	50^#^	50^#^	70^*^	80^*^
Arrhythmia incidence (%)	45	35^*^	35^*^	30^*^	20^*^
Dopamine (mg)	687 ± 63	513 ± 71^∗#^	478 ± 67^∗#^	317 ± 46^*^	243 ± 54^∗#^
Sodium nitroprusside (mg)	813 ± 64	796 ± 66^#^	749 ± 81^#^	706 ± 83^*^	641 ± 83^∗#^
ICU stay (h)	68.6 ± 9.1	62.0 ± 8.9^∗#^	59.1 ± 8.8^∗#^	54.5 ± 8.9^*^	43.1 ± 9.3^*^
Hospital stay (d)	16.2 ± 2.5	14.69 ± 2.9^∗#^	13.2 ± 2.8^*^	12.8 ± 2.1^*^	10.3 ± 1.6^∗#^
Death in hospital (*n*)	0	0	0	0	0

Control: untreated control; Cap1hr: captopril treatment for 1 h before surgery (12.5 mg, p.o., 1 h before surgery); Cap3d: captopril treatment for 3 days (12.5 mg, p.o., 3 times a day) before surgery; Cap1hr+Iso: captopril treatment for 1 h in combination with inhalational isoflurane (Iso) at 1.1% end tidal concentration administrated for 30 min before CPB; Cap3d+Iso: captopril treatment for 3 days in combination with Iso before CPB. Values are means ± S.D. or the number of patients. ^*^ *P* < 0.05 versus Control, ^#^ *P* < 0.05 versus Cap1hr+Iso.
